# Enhancing reservoir characterization in the Temsah gas field through high-resolution seismic analysis and three-dimensional modeling

**DOI:** 10.1038/s41598-025-22108-w

**Published:** 2025-12-09

**Authors:** Mohamed Reda, Nader H. El-Gendy, Mohamed I. Abdel-Fattah, Mohamed M. Elmashaly, Moataz Kh. Barakat

**Affiliations:** 1 Faculty of Science, Geology Department, Al-Azhar University, Nasr, P.O. Box 11884, Cairo, Egypt; 2 Geology Department, Faculty of Science, Tanta University, Tanta, 31527 Egypt; 3 Petroleum Geosciences and Remote Sensing Program, Department of Applied Physics and Astronomy, College of Sciences, University of Sharjah, Sharjah, 27272 UAE; 4 Geology Department, Faculty of Science, Suez Canal University, Ismailia, Egypt; 5 ECS Electronic construction service, Kattamia, Kattamia, P.O. Box 8, New Cairo, Egypt

**Keywords:** Seismic data interpretation, 3D structural model, Reservoir model, Petroleum system element, Prospect estimation, Offshore nile delta, Geology, Geophysics, Solid Earth sciences

## Abstract

The offshore Temsah Gas Field, located about 65 km NNW of Port Said in the northeastern Nile Delta Basin, is structurally complex, with NE–SW and NW–SE normal faults that control reservoir compartmentalization and hydrocarbon entrapment. The Sidi Salem Formation, the primary reservoir, comprises interbedded sandstone and shale facies with significant hydrocarbon potential. This study integrates high-resolution post-stack time-migrated 2D seismic data and well log analysis from four wells and twenty-nine 2D seismic lines to delineate reservoir structures, evaluates the hydrocarbon potential of the Sidi Salem Formation, and builds a 3D geological model to enhance field productivity, while also clarifying fault geometries, quantifying key petrophysical parameters, and pinpointing new exploration prospects. Seismic interpretation reveals a prominent horst block with a three-way dip closure and several fault-bounded traps. Petrophysical analysis indicates net reservoir thickness of 22–120 m, effective porosity of 19–34%, shale content of 8–27%, and hydrocarbon saturation of 70–79%. Integration of seismic and petrophysical data delineates sandstone-rich zones with enhanced reservoir quality, mainly within the upthrown fault blocks. The resulting 3D model supports volumetric estimation, identifying a new fault-bounded prospect with an estimated GIIP of ~5.33 TCF. This integrated workflow reduces structural uncertainty, refines reservoir characterization, and offers a reproducible approach for exploration in fault-controlled deltaic reservoirs.

## Introduction

Clastic reservoirs in sedimentary basins are key targets for hydrocarbon exploration and production, as they store large volumes of oil and gas. Advances in seismic imaging and drilling technologies, particularly in the Nile Delta Basin, have greatly improved reservoir identification and extraction. These reservoirs remain crucial for both conventional and unconventional energy resources. The Nile Delta Basin, one of the largest deltaic basins worldwide, spans approximately 250,000 square kilometers^1^. Its subsurface sedimentary succession exceeds 6.0 kilometers in thickness and comprises deposits ranging from the Oligocene to the Quaternary periods^2–4^. The Hinge Zone delineates the southern boundary of the rifted zone and trends east–west.

**Fig. 1:**
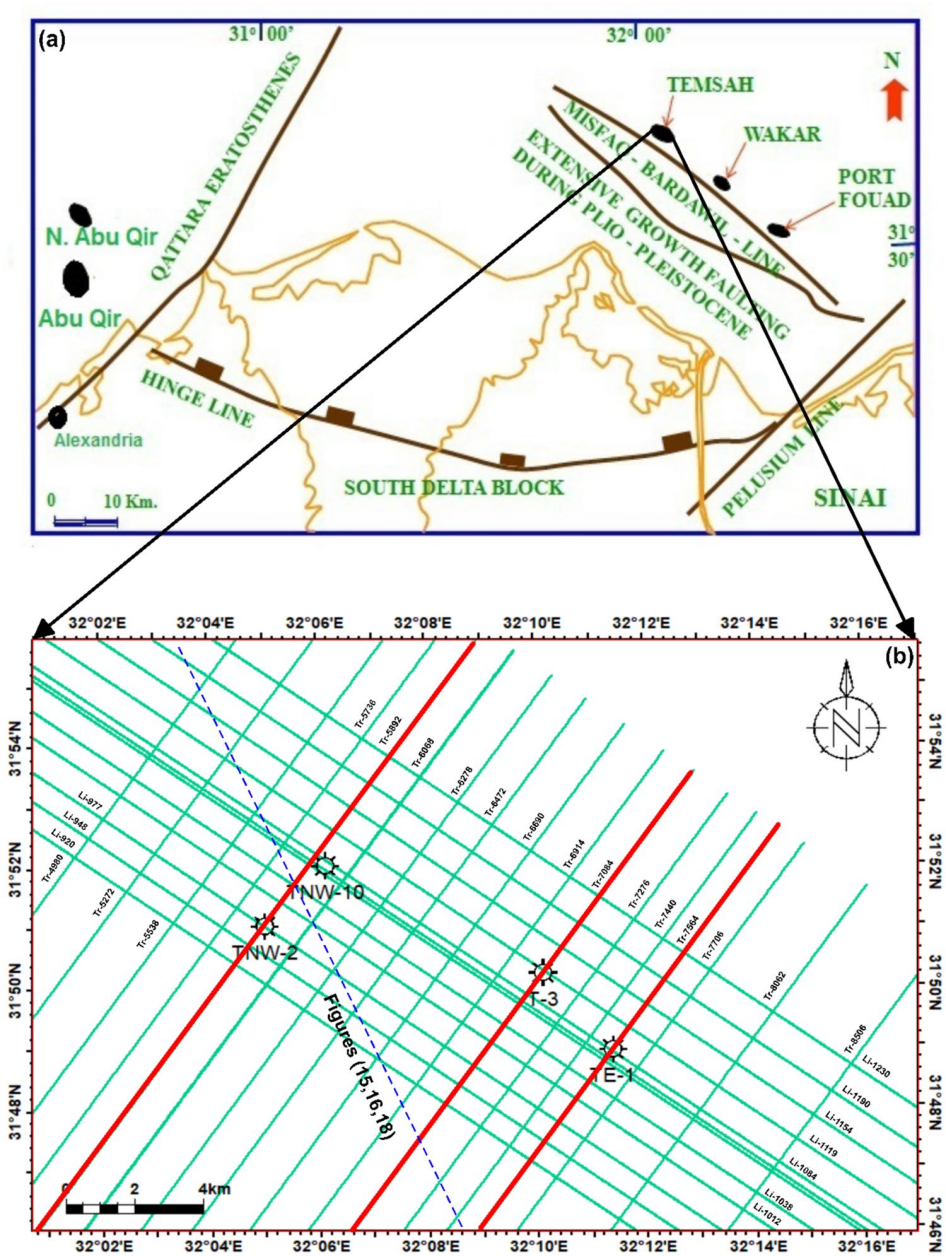
**a** Regional structural framework of the northern Nile Delta showing major fault trends and tectonic elements that influence hydrocarbon accumulation (after^17,18^). **b** Location map of the Temsah Field with studied wells and 2D seismic lines. The red line indicates the orientation of the interpreted seismic profiles (created in Petrel, 2017).

The Nile Delta occupies the northern margin of the NE-African Plate, extending from the Cretan and Cyprus subduction arcs to the Red Sea^5–9^. Tertiary reservoirs overlie the pro-delta shales of the Qantara Formation^10,11^. Serravallian–Tortonian progradation generated prolific gas and condensate accumulations, including the Temsah Field, which exhibits both structural and stratigraphic traps^12,13^. The offshore Nile Delta Basin is a globally significant hydrocarbon province, with reservoirs ranging from the early Oligocene to early Pliocene^14–16^. The study area is the Temsah Concession (Fig 1), located ~65 km north of the Mediterranean coastline and covering ~400 km^2^. The Sidi Salem Formation, the primary reservoir, exhibits significant variability in facies distribution, which directly affects reservoir quality and gas accumulation.

Petrophysical analysis and high-resolution seismic interpretation are fundamental for reservoir characterization and prospect identification^19,20^. Petrophysical evaluation quantifies porosity, shale content, and fluid saturation, enabling the delineation of pay zones, assessment of storage capacity, and discrimination of lithologies such as sandstone, shale, and limestone. Seismic interpretation maps faults, folds, and stratigraphic features, constraining the structural framework and depositional architecture that control hydrocarbon distribution. Integrating these datasets yields a comprehensive understanding of reservoir properties, heterogeneity, and fluid distribution, thereby reducing uncertainty and guiding exploration and development strategies.

Three-dimensional (3D) static modeling provides a vital link between petrophysical analysis and seismic interpretation, enabling detailed characterization of reservoir architecture, heterogeneity, and fluid distribution^21–25^. This integration supports reliable reservoir simulation and informed field development planning. In the Temsah Field, however, fault geometry and reservoir heterogeneity remain uncertain due to reliance on 2D seismic data and limited well control^10^. This study addresses these challenges by combining high-resolution seismic interpretation with advanced 3D static geological modeling to refine the structural framework and reservoir characterization. Earlier studies in the Temsah area primarily emphasized structural mapping, stratigraphic framework definition, and prospect evaluation using conventional 2D seismic and well-log data^16^.

The objective of this study is to integrate petrophysical analysis and high-resolution seismic interpretation to evaluate the Neogene–Quaternary succession—particularly the Sidi Salem Formation—of the Temsah Field in the eastern offshore Nile Delta. Reprocessed 2D seismic data, tied to well logs, provide improved frequency bandwidth and signal-to-noise ratio, enabling precise fault and horizon mapping and reducing structural uncertainty. The resulting 3D static model captures spatial facies variation, reservoir architecture, and key petrophysical parameters, supporting the identification of drilling targets and optimization of field development strategies. This integrated approach delivers a robust understanding of subsurface conditions, guiding reservoir management and hydrocarbon recovery decisions.

The novelty of this study lies in its integrated workflow, combining high-resolution seismic interpretation, detailed petrophysical analysis, and advanced 3D static modeling to resolve uncertainties in fault geometry and reservoir heterogeneity within the Sidi Salem Formation. This approach enables precise delineation of fault-bounded traps, prediction of spatial reservoir quality variations, and identification of a new gas-bearing prospect with significant estimated reserves. The methodology enhances the geological understanding of the Temsah Field and provides a reproducible framework for characterizing structurally complex, deltaic hydrocarbon provinces.

## Geological setting and stratigraphic framework

### Stratigraphic setting

**Fig. 2:**
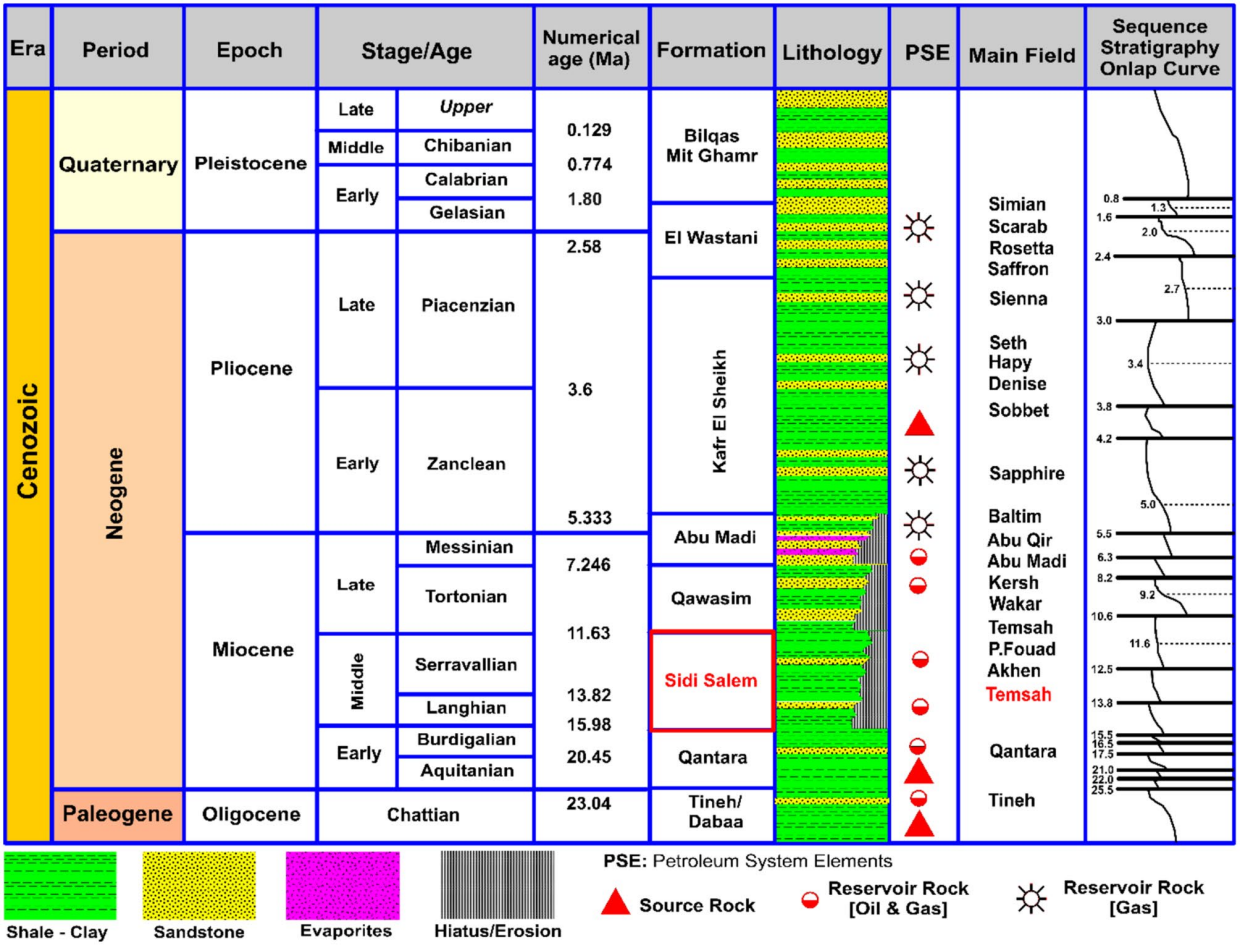
Comprehensive stratigraphic column of the Nile Delta Basin illustrating the main lithostratigraphic units, depositional environments, and reservoir–seal relationships of the study area (after^36^).

The stratigraphic column of the Nile Delta can be succinctly summarized as: the Paleozoic stratigraphic units are situated in the eastern, southern, and western regions of the Delta, where the Nile Delta area was once covered by the Paleozoic Sea. The Paleozoic sedimentary sequence is expected to extend across the entirety of the Nile Delta region, completely overlying the basement rocks, with an estimated thickness ranging between 750 and 1500 meters^26,27^. During the final stages of the Paleozoic era, there were geological movements in specific areas that led to the gradual wearing a way of most of the sediments that had been deposited before the Carboniferous period. The Mesozoic stratigraphic units, consist primarily of carbonate or marl deposits from a shelf habitat. Two significant unconformities are worth mentioning: the Cimmerian unconformity, which happened during the Late Kimmeridgian period and was caused by a global drop in sea level and the closing of the Paleo-Tethys Ocean, and the unconformity that occurred during the latest Maastrichtian to early Paleocene period, following the uplift of the Syrian Arc. The Pre-Miocene Formations, which consist of a series ranging from the Upper Jurassic to the Oligocene. The deposits are composed of typical sediments found near the shore, including shelf and lagoonal deposits^28–33^. The depositional history of the Nile Delta may be traced back to the Jurassic period, which is when the oldest accessible data is referenced^34^. The earliest Mesozoic Formation found in the Delta is from the Late Jurassic period. The Late Mesozoic deposits exhibit a generally regressive shoreline environment, characterized by notable unconformities at both the upper and lower boundaries of the series^35^. The Paleogene (Tertiary) stratigraphic units often rest on top of Late Cretaceous or earlier strata with an unconformity. They contain a conglomerate within the same formation, which contains fossils from the Late Cretaceous period that have been eroded and redeposited^35^. During the late Paleocene to early Eocene period, there was a transgression in the area. However, due to the high elevation of the general area, only a little amount of material was brought ashore. This resulted in the deposition of a thin sequence of mostly siliceous limestones, primarily in localized low areas. The sedimentation throughout the late Eocene to early Oligocene period resulted in the deposition of deep marine marls that gradually transitioned into shale stratigraphic horizons/units. This sequence represents a transition from the deposition of carbonate sediments to the deposition of terrigenous sediments. The sediments from the Oligocene to the earliest Miocene period are found in a maritime environment and are distinguished by the northward expansion of shelf sediments and the release of turbiditic flows. During the Aquitanian-Burdigalian time a substantial series of deep-sea marls and shales with smaller amounts of coarse terrigenous sediments were laid down in the delta region (known as the Qantara Formation). The Langhian and Serravallian periods were distinguished by a notable pace of sinking and a substantial influx of sediment, resulting in the construction of a substantial terrigenous sequence known as the Sidi Salim Formation (Fig 2).

The onshore area exhibits a transition from southern to northern deposits, gradually changing from transitional to shallow marine sediments. Further out in the shelf, there is a predominance of finer sand and shale. This gives way to a predominantly shaly prograding complex, with turbiditic sandy systems being deposited towards the basin, particularly in the Temsah and Wakar areas. During the Serravallian period, a significant erosive event occurred because of a sequence of upward movements. This event is documented over the entire area by a notable unconformity known as the Serravallian/Tortonian unconformity. The Tortonian period, like the preceding Serravallian period, is characterized by a northward prograding shelf system. In its frontal area, a turbiditic complex is deposited, specifically the Sidi Salim Formation in the Baltim area and the Wakar Formation in the Temsah and Wakar areas. Compared to the Serravallian sequence, the Tortonian sequence is distinguished by a shift of the depositional systems towards the basin, indicating an overall regressive trend^26^.

### Structural setting

**Fig. 3:**
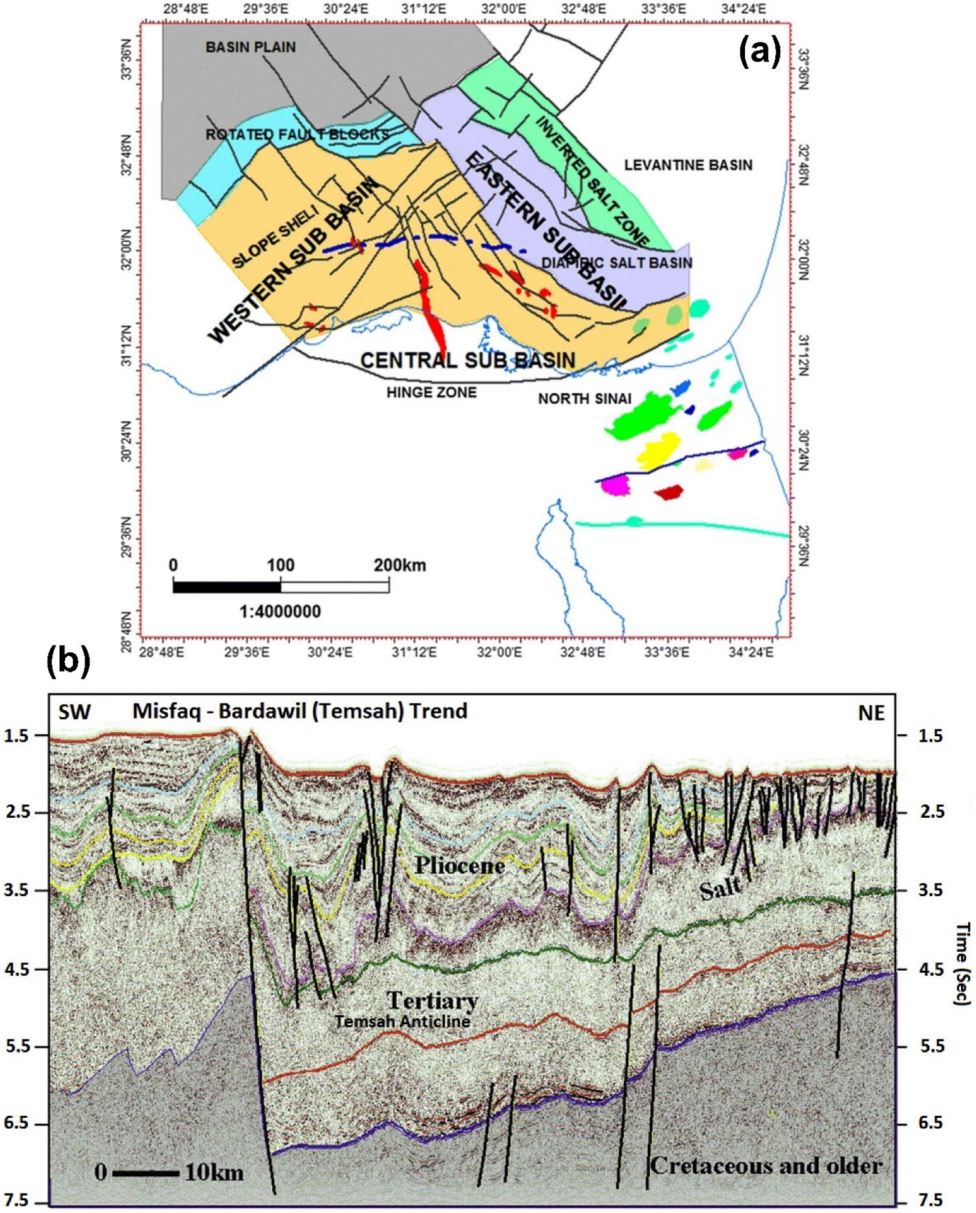
**a** Major structural elements of the Nile Delta, with red zones representing the main producing fields (after^43,44^). **b** Regional NE–SW seismic line across the Temsah fault trend, highlighting the structural complexity and main trapping configurations (after^45^).

The Temsah Gas Field, a key player in Egypt’s gas production, is shaped by regional tectonics and stratigraphy, which strongly influence the Sidi Salem reservoir. Normal faults trending NE-SW and NW-SE create structural traps, while depositional variations control facies distribution. Depth mapping and 3D static geological model highlight a horst block with a three-way dip closure and a step fault as key hydrocarbon trap. Increased sandstone content in the northwestern and southeastern regions enhances reservoir quality, with higher porosity and hydrocarbon saturation. These geological controls are essential for guiding future exploration and drilling in the field. Three principal fault types have been identified within the structural framework of the Nile Delta^37–42^: Major faults, Pre-Messinian faults, and Post-Miocene faults. These structural elements are predominantly located in the offshore region of the delta. (Fig 3).

1. Major faults

The primary faults have been consistently active, to different extents, from the Miocene epoch until the present. Three prominent fault systems were identified in the offshore Nile Delta region. The first fault system extends in a northwest-southeast direction, with a downward displacement towards the northeast. It is situated to the north of Damietta. The second trend is oriented in a northeast-southwest direction and exhibits a downward dis-placement towards the northwest. The third fault is oriented in an east-west direction and has a northward dip. It is situated to the north of Alexandria. The hinge zone, which runs in an east-west direction, is composed of a sequence of step faults that dip towards the north (forming a flexure zone with a width of 30–40 km). These faults are associated with the crustal rupture that occurred during the Jurassic period. The hinge zone delineates the interface between a stable platform to the south (South Delta block) and a subsided basin to the north, characterized by thicker and relatively deep marine successions in all Cenozoic sequences^30^.

(2) Pre-messinian faulting

This faulting comprises of little faults, whose orientations are challenging to determine, perhaps because of the diapiric influences. The faulting that occurred after the Miocene period was caused by gravity, leading to the formation of slump structures and listric fault^1,46–48^. The Tethyan trend is an east-west geological feature that may be associated with the initial separation of the southeastern Mediterranean continental margin during the early Mesozoic era, and possibly much earlier. Prominent illustrations of this phenomenon are the Oligo-Miocene Hinge Zone, Mit Ghamr Fault, and the northern and southern bends of the onshore Nile Delta. The Rosetta trend is a geological feature that extends in a northeast-southwest direction. During the Late Cretaceous period, notable geological features include the Pelusium, Qattara – Eratosthenes, as well as the Gamasa, Idfina, and Port Said-Hout lines. It is theorized that these faults originated from a singular point in the northeastern region of the Mediterranean Sea, particularly near Alexandria^49,50^. These faults exhibit both vertical movement and sinistral strike-slip displacement.

(3) Post-miocene faulting

The Miocene period was characterized by a northwest–southeast trend that remained active. The most renowned example is the Temsah or Bardawil Line in the eastern offshore Nile Delta^51^. Most of the faults have a predominant north 45°W orientation. However, a few faults align with the Gulf of Suez and Red Sea geological trend, approximately north 30°W.

## Data and methods

### Data

This study utilized a combination of well-log and seismic datasets acquired from the offshore Temsah Gas Field in the northeastern Nile Delta Basin.

- **Well data:** Four wells—TE-1, T-3, TNW-10, and TNW-2—were selected for detailed analysis. Available open-hole logs included gamma ray (GR), sonic (DT), density (RHOB), neutron porosity (NPHI), and resistivity curves. Formation top markers, lithostratigraphic descriptions, and production reports provided by the operating companies were also incorporated.
- **Seismic data:** Twenty-nine post-stack time-migrated 2D seismic reflection profiles, oriented predominantly NE–SW and NW–SE, were available for interpretation. These data were provided in SEG-Y format with appropriate navigation files. The seismic dataset consists of 29 high-resolution post-stack time-migrated 2D lines with a dominant frequency of approximately 30–35 Hz, providing a vertical resolution of ~20–25 m in the depth range of the Sidi Salem Formation. The reprocessing included pre-stack time migration, coherent noise suppression, deconvolution, and spectral whitening to broaden the usable frequency bandwidth. These steps significantly improved reflector continuity, fault delineation, and the ability to resolve thin stratigraphic units, thereby increasing the accuracy of structural and stratigraphic interpretation.
- **Software and tools:** Seismic and geological interpretations were performed using Petrel 2017 (Schlumberger), while petrophysical analysis was conducted with Interactive Petrophysics (IP) 2018. Microsoft Excel was used for volumetric calculations, and all datasets underwent rigorous quality control to ensure consistency and reliability.

### Methods

**Fig. 4:**
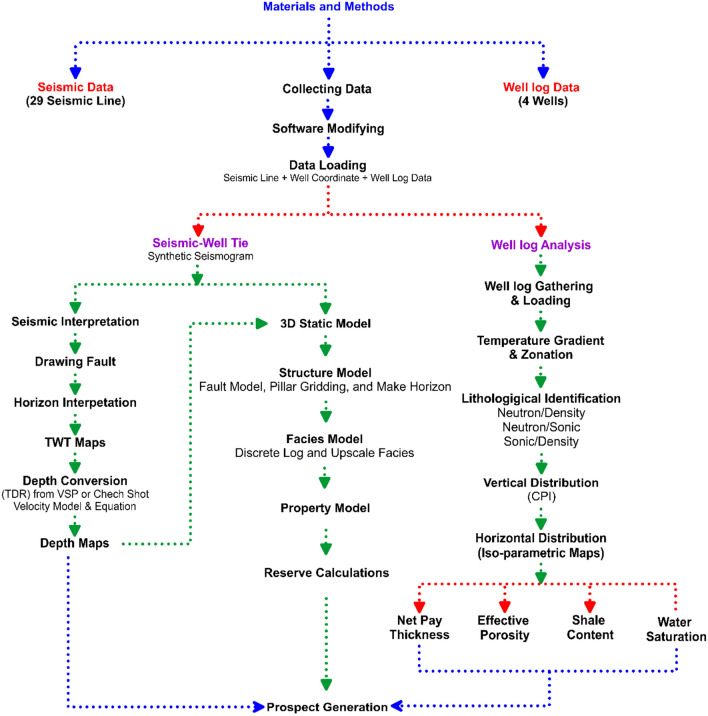
Workflow illustrating the integrated methodology of this study, including well-log analysis, seismic interpretation, 3D structural and property modeling, and volumetric estimation for prospect evaluation.

The methodological workflow was designed as an integrated process comprising four main stages (Fig 4):

- **Step 1: well log analysis**

Well logs from four key wells were processed in Interactive Petrophysics to determine effective porosity, shale volume, water saturation, and net-to-gross ratio. Lithofacies classification (sandstone vs. shale) was conducted using cross-plot and cut-off methods, with cutoff values typically applied in the Temsah Gas Field in the Nile Delta Basin: effective porosity ≥ 10%, shale volume ≤ 30%, and water saturation ≤ 65% for hydrocarbon-bearing intervals.

- Step 2: seismic interpretation

Seismic data interpretation is a key process in geophysics and petroleum exploration, enabling the delineation of subsurface geological structures and potential hydrocarbon reservoirs. Seismic horizons, observed as distinct reflectors, are interpreted to identify faults, channels, reservoir bodies, and other stratigraphic and structural features. Integration with well data helps validate and correlate interpreted horizons with subsurface rock properties^46–50^. In this study, post-stack time-migrated 2D seismic lines (n = 29) were imported into Petrel for detailed interpretation. Faults and horizons were manually picked and validated based on seismic waveform characteristics (Fig 1b). Formation top data from wells TE-1, T-3, TNW-2, and TNW-10 were tied to seismic sections to establish stratigraphic correlations and link seismic reflections with lithostratigraphic units^52–54^. Well-to-seismic ties were performed using synthetic seismograms generated from sonic and density logs. This process ensured accurate alignment between seismic reflectors and formation tops, reducing uncertainty in horizon picking and improving the reliability of the structural framework. Interpreted horizons were subsequently converted to both structural time and depth contour maps to analyze fault geometries, closure configurations, and overall structural patterns.

- Step 3: three-dimensional static reservoir modelling

Constructing a three-dimensional static model is a critical stage in reservoir characterization and field development planning. It involves integrating geological, geophysical, and petrophysical datasets—such as well logs, seismic data, and production records—into a coherent representation of the subsurface reservoir. All input datasets are carefully evaluated for quality and suitability prior to modeling^55–57^. The modeling process in this study comprises two main stages: structural modeling and property modeling.

- Structural modeling

Following seismic interpretation, a 3D structural framework was constructed using pillar gridding in Petrel. Key horizons, faults, and other structural features were incorporated based on the interpreted seismic data and structural geological concepts^58–63^. The structural modeling process consisted of three steps:

1. **Fault modeling:** Characterization of the fault network affecting the formations under investigation, including geometry, orientation, and displacement.
2. **Framework gridding:** Creation of the structural grid framework to represent the overall reservoir architecture.
3. **Horizon modeling:** Delineation and classification of key horizons, followed by subdivision into stratigraphic units.

This stratigraphic subdivision allowed for the identification of structural influences on reservoir geometry, lateral and vertical facies variations, and changes in petrophysical properties, particularly within the Sidi Salem reservoir^64–68^.

- Property modeling

Property modeling was performed to estimate the geostatistical distribution of lithofacies and petrophysical properties within the defined stratigraphic units^69–73^. Well-log and seismic-derived interpretations were integrated to define and map key horizons, capture lithological variations, and model facies distribution. The process included:

1. Facies modeling: Classification and spatial modeling of lithofacies using well data and geological trends.
2. Well upscaling: Aggregation of fine-scale log data into the structural grid while preserving key flow-related properties.
3. Petrophysical property modeling: Application of geostatistical algorithms to generate 3D models of porosity, permeability, water saturation, and shale content.

Reservoir heterogeneity and anisotropy were explicitly incorporated to ensure the static model realistically represents subsurface conditions. The final integrated model forms the basis for volumetric estimation and subsequent dynamic reservoir simulation.

- Step 4: hydrocarbon volumetric calculation

Petrophysical parameters from Step 1 were integrated into the 3D structural model to generate property models (porosity, Vsh, Sw). Gross rock volume (GRV) was calculated for identified structural closures, and gas initially in place (GIIP) was estimated using the standard volumetric equation, enabling the identification of the most prospective zones for drilling. Hydrocarbon volumes were estimated following the method described by^74^, using the formula:

1 $${\text{HCIIP}} = \, \left( {{\text{GRV}}*\phi {\text{ NTG}}*\left( {{1} - {\text{S}}\_{\text{w}}} \right)} \right) \, /{\text{FVF}}$$

Where: HCIIP = Hydrocarbons Initially In Place, GRV = Gross Rock Volume, ϕ\phi = Porosity, NTG = Net-to-Gross Reservoir Ratio, _Sw​ = Water Saturation, and FVF = Formation Volume Factor. The gas initially in place (GIIP) and oil reserves were subsequently calculated based on this HCIIP volume for the Sidi Salem Formation.

The methodological workflow was specifically designed to address the fault-controlled structural complexity and facies heterogeneity of the Sidi Salem Formation. The integration of these datasets into a 3D static model allowed for the accurate delineation of high-quality reservoir zones and the identification of a new fault-bounded prospect. This tailored approach ensures the methods are directly applicable to structurally complex deltaic reservoirs with similar tectono-stratigraphic settings.

This study is limited by the availability of well data, as only four wells were analyzed, which may not fully capture reservoir heterogeneity. Additionally, the reliance on 2D seismic data restricts the resolution of structural interpretations compared to 3D seismic imaging. Variability in petrophysical parameters and uncertainties in depth structure mapping could also impact the accuracy of the 3D geological model. Further studies incorporating additional wells and high-resolution 3D seismic data would enhance the reliability of reservoir characterization and hydrocarbon potential assessment.

## Results and discussion

### Stratigraphic interpretation

The stratigraphic correlation approach is a method employed to establish a connection between distinct rock stratigraphic horizons/units or strata in various geographical regions. The objective is to ascertain whether the strata in one region are comparable or analogous to those in another region, and if so, to establish their chronological order and depositional chronicles in relation to each other. The initial stage in constructing a three-dimensional representation of oil or gas reservoirs involves ascertaining the stratigraphic placement of the designated region to comprehend the prevailing stratigraphic sequence and the interrelationships among the various stratigraphic horizons/units. The identification of unconformity surfaces is crucial for understanding the genesis of significant stratigraphic petroleum reserves, thereby necessitating precise indication of their existence or absence. The focus of this study is to the Middle Miocene, with particular emphasis on the Sidi Salem Formation. This specific formation serves as the primary reservoir in many fields located in the Nile Delta of Egypt^38–40^. The substantial thickness and unique compositional characteristics of this stratigraphic horizons/units contribute to its significance in hydrocarbon exploration.

**Fig. 5:**
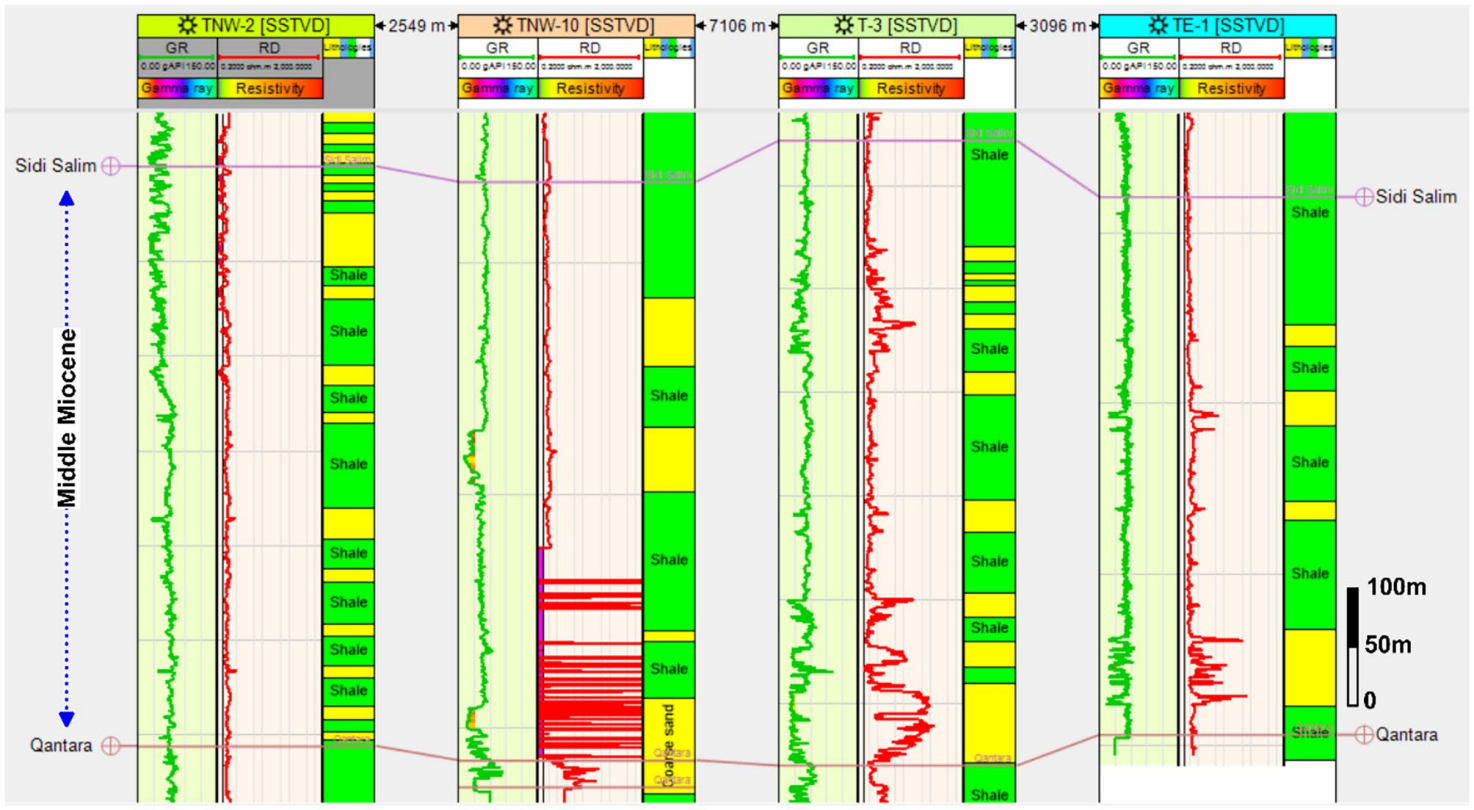
NW–SE lithostratigraphic correlation between the studied wells, showing lateral continuity and vertical variation within the Sidi Salem Formation. The section highlights thickness changes, facies variability, and reservoir heterogeneity across the Temsah Field.

To facilitate a comparative analysis of the Sidi Salem Formation in the wells under investigation (namely, TE-1, T-3, TNW-2, and TNW-10), a vertical correlation section was created. This correlation was placed in a left-to-right sequence, with TNW-2 being the left-most well and TE-1 being the rightmost well. The goal of this arrangement was to enable a comprehensive examination and comparison of the Sidi Salem Formation (Fig5). The Sidi Salem Formation is stratigraphically underlining the Messinian Formation, forming an unconformity connection. It is distinguished by its considerable thickness, primarily focused on the northwestern direction and in the central part of the Temsah field through TNW-2 and TNW-10 wells. The net reservoir thickness steadily diminishes in the southeast direction. The correlation clearly indicates that the TE-1 well has the lowest net reservoir thickness of the Sidi Salem Formation. Specifically, the measured thickness in TE-1 is approximately 22 meters, which is significantly lower compared to the thickness range of 85–120 meters observed in the other studied wells.

The Sidi Salem Formation exhibits variable thickness across different locations: approximately 1000 meters in the northern Delta embayment^41^, and notably thicker, ranging from 1200 to 1670 meters in the offshore Nile Delta^42^. Identified as Tortonian-Serravallian in age^43^. it is characterized by unconformable deposition over the Qantara Formation and/or marine Oligocene or older sediments^12^. Its upper boundary is marked by a thick sandy conglomeratic bed, and it is unconformably covered by the Qawasim Formation, with offshore regions further overlain by the lower Abu Madi Formation^31,32,42^. Sedimentary features suggest diverse deposition environments, with shales indicating foreshore to deep marine conditions during transgressive phases, while sandstones suggest regressive periods^33^. Significant lateral and up-dip facies changes are noted within the Sidi Salem Formation and the middle Miocene sequence overall^46^, possibly originating from nearby igneous and metamorphic rocks, such as those in the Red Sea hills.

The observed thinning of the Sidi Salem Formation towards the southeast is interpreted to result from both depositional and structural influences. Increased shale content in this sector, coupled with its position adjacent to fault-bounded structural highs, suggests deposition in a more distal, low-energy environment, which favoured fine-grained sediment accumulation over sand-rich facies.

### Inferred results from interpretation

Seismic interpretation of the Sidi Salem Formation reveals a structurally complex framework controlled by NE–SW and NW–SE trending normal faults. These faults form multiple fault-bounded closures, with a prominent horst block in the central field exhibiting a three-way dip closure, identified as the main hydrocarbon trap. Depth structure mapping highlights additional step-fault closures along the southeastern sector.

The fault orientations (strike) in the study area were identified based on seismic interpretation; however, dip angles were not explicitly detailed in the initial analysis. Recognizing the importance of fault dip for defining the geometry and volume of fault-bounded blocks, subsequent interpretations included an assessment of dip values derived from seismic dip measurements and well data. These dips vary across the fault trends, generally ranging between 60° and 80°, which significantly affect the shape and size of horst and graben structures controlling reservoir compartments. Incorporating fault dip improves the three-dimensional understanding of the structural framework and enables more accurate reservoir volume estimations critical for hydrocarbon exploration and development.

Petrophysical analysis from four key wells indicates effective porosity values ranging from 19% to 34%, shale content between 8% and 27%, and hydrocarbon saturation from 70% to 79%. Net reservoir thickness varies between 22 m and 120 m, with the TE-1 well recording the lowest thickness. The highest-quality zones are concentrated in the northwestern and southeastern parts of the field, where increased sandstone content correlates with higher porosity and gas saturation. The integration of seismic and well-log data into a 3D static model highlights pronounced spatial lithological variability, with sandstone-rich zones forming preferential gas-bearing intervals. This modeling approach delineates a newly identified, fault-bounded gas prospect within the Sidi Salem Formation, with an estimated GIIP of 5,332,660 BCF.

**Fig. 6:**
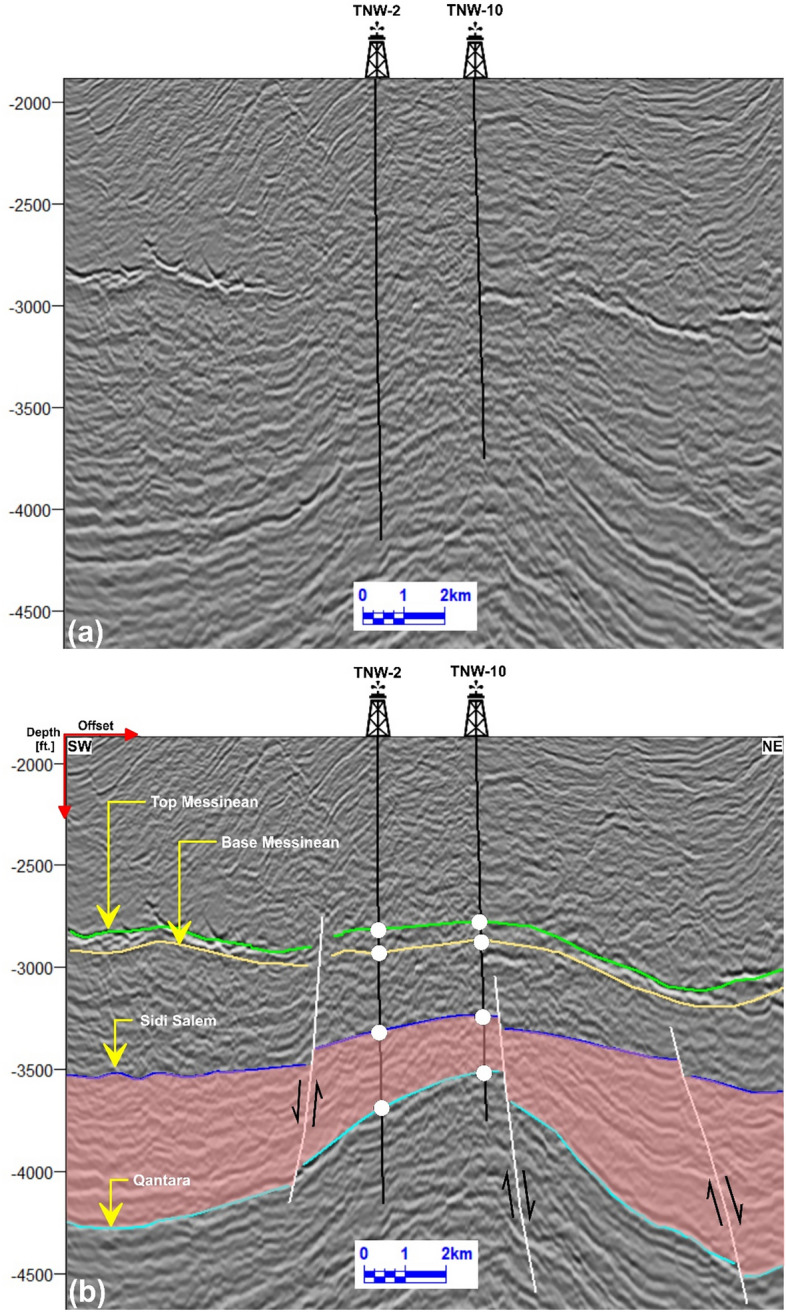
**a** Uninterpreted seismic line Tr-5892. **b** Interpreted NE–SW seismic line Tr-5892, showing three normal faults and their impact on the Sidi Salem Formation. Wells TNW-2 and TNW-10 are located on the uplifted side of the faults, corresponding to favorable structural closures.

**Fig. 7.**
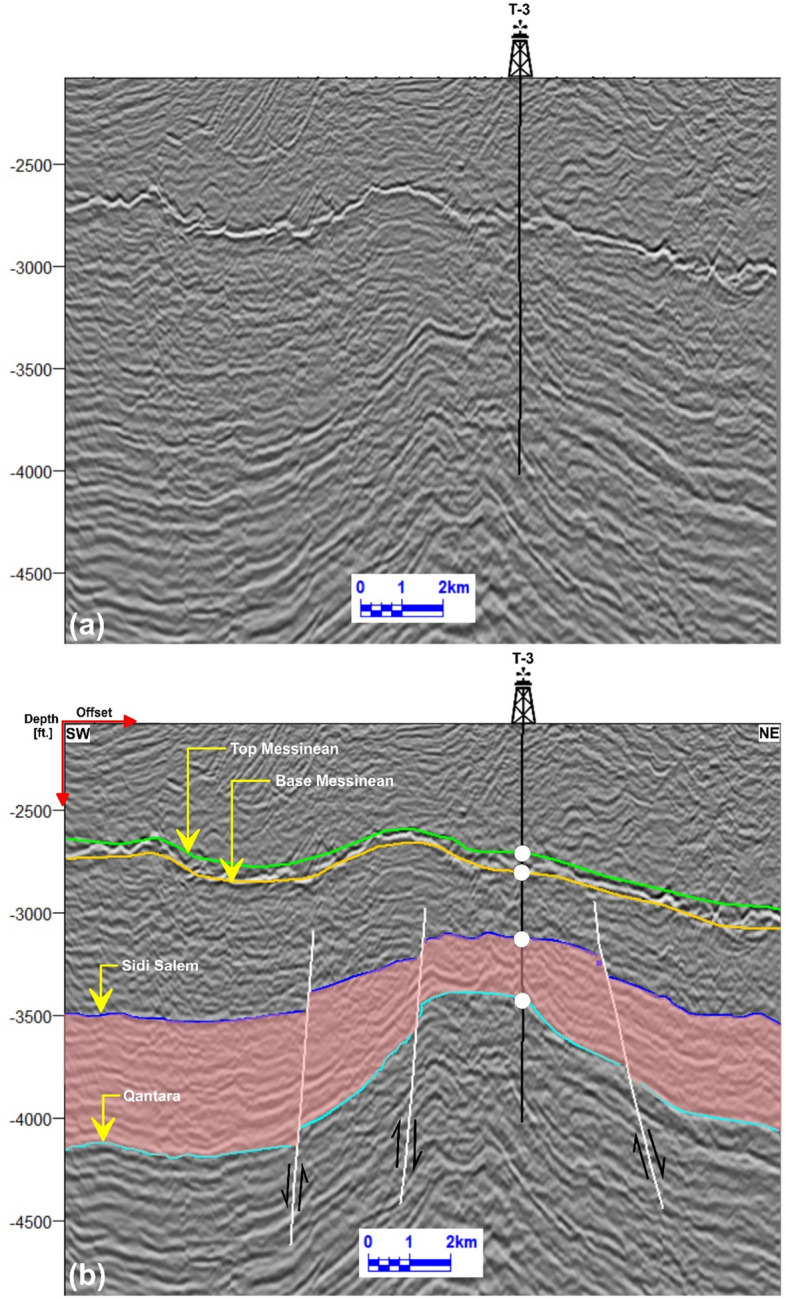
**a** Uninterpreted seismic line Tr-7084. **b** Interpreted NE–SW seismic line Tr-7084, illustrating a cluster of step and horst faults affecting the Sidi Salem reservoir. The T-3 well penetrates the uplifted block, confirming reservoir presence in structurally elevated positions.

**Fig. 8:**
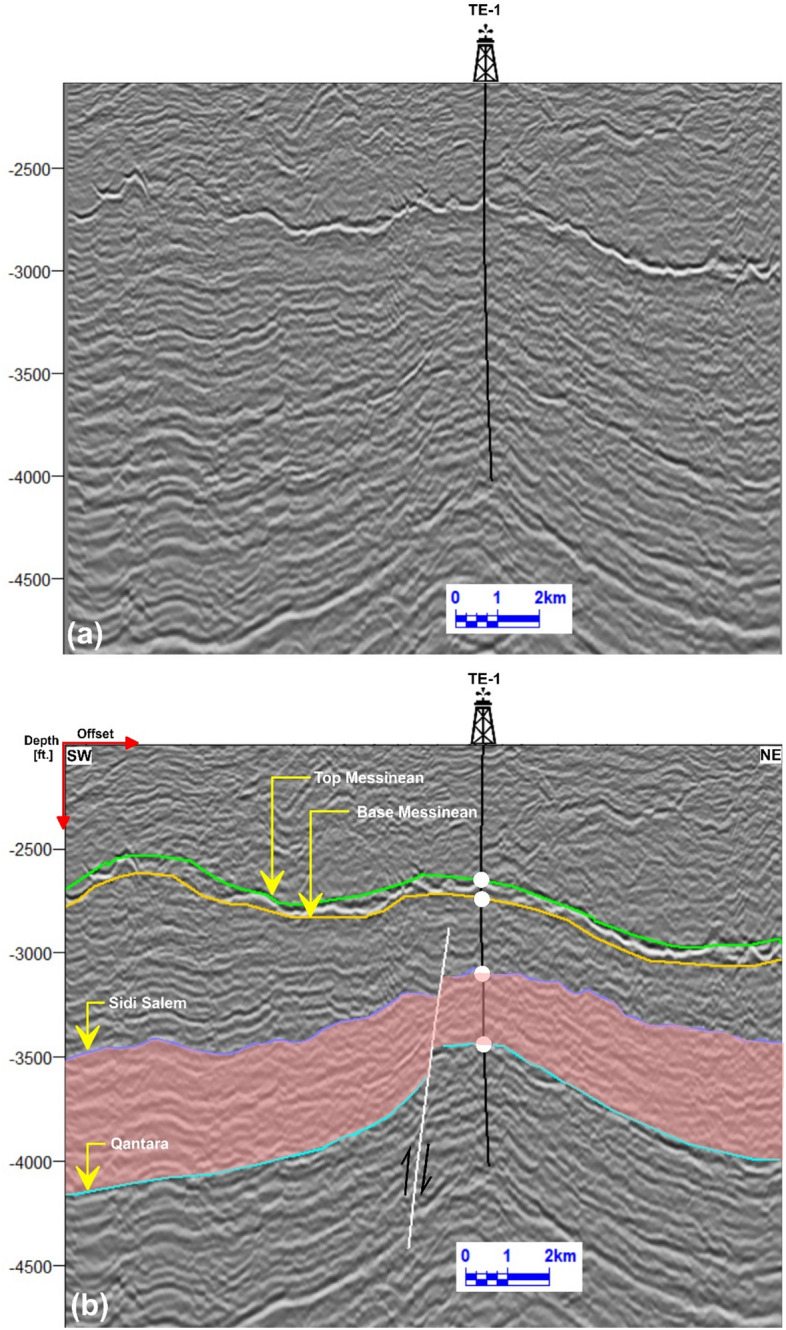
**a** Uninterpreted seismic line Tr-7564. **b** Interpreted NE–SW seismic line Tr-7564, crossing the southeastern Temsah Field. The seismic profile reveals step-fault geometry, with the TE-1 well drilled in the uplifted block of the Sidi Salem Formation.

Seismic data interpretation, crucial for understanding subsurface geology, was performed on twenty-nine reflection profiles (Fig 1b), focusing on both structural and stratigraphic features. Among these, three key seismic lines (Tr-5892, Tr-7084, and Tr-7564), oriented northeast-southwest and intersecting wells TNW-2, TNW-10, T-3, and TE-1, were analyzed in detail (Figs 6–8). The study emphasized the Messenian, Sidi Salem, and Qantara Formations to characterize their seismic responses and evaluate fault impacts. The well-to-seismic tie, marked by white circles on seismic sections, was established to enhance correlation accuracy by aligning data with seismic horizons based on formation tops^4,75–77^).

Fig 6 illustrates the northwest-southeast interpreted seismic line named Tr-5892 which is near the northwestern portion of the study area. They are intersecting with the TNW-10 and TNW-2 wells. The study area, which focuses on the Sidi Salem Formation as the main reservoir, appears to be affected by three normal faults that trends in the north-east-southwest direction. The downthrown side of this fault is oriented towards the northwest. The Sidi Salem Formation is expected to produce in all these wells because they are drilled on the uplifted side, forming a favourable closure for oil and gas entrapment.

Fig 7 shows the NE-SW interpreted seismic line (Tr-7084), which is in the central part of the study area passing through T-3 well. The seismic line reveals that the upper section of the study area, primarily associated with Sidi Salem Formation, is influenced by a cluster of three normal faults, forming a group of step and horst faults blocks. The T-3 well is drilled in the uplifted side of these normal faults. The interpreted faults intersect all the formations under investigation.

Fig 8 presents the interpretation of a north-east-southwest seismic line known as Tr-7564. This seismic line crosses the southeastern region of the Temsah field and intersects with the TE-1 well. In this line, the area is influenced by one normal fault that trend in the northeast-southwest direction. The down-thrown side of these faults is oriented towards the northeast. This normal fault creates a step fault blocks, affecting all the formations studied, particularly the Sidi Salem Formation. The results suggest the existence of three normal faults, which have led to the creation of horst, and step faults in the Temsah field. In addition, the analyzed wells successfully drilled into the Sidi Salem Formation, as shown in the previous figures. To visually depict the subsurface structure in relation to its depth, it is crucial to generate a map that illustrates the structure at various depths. Geoscientists can improve their understanding of the intricate geological architecture under the surface by creating a comprehensive depth structure map.

**Fig. 9:**
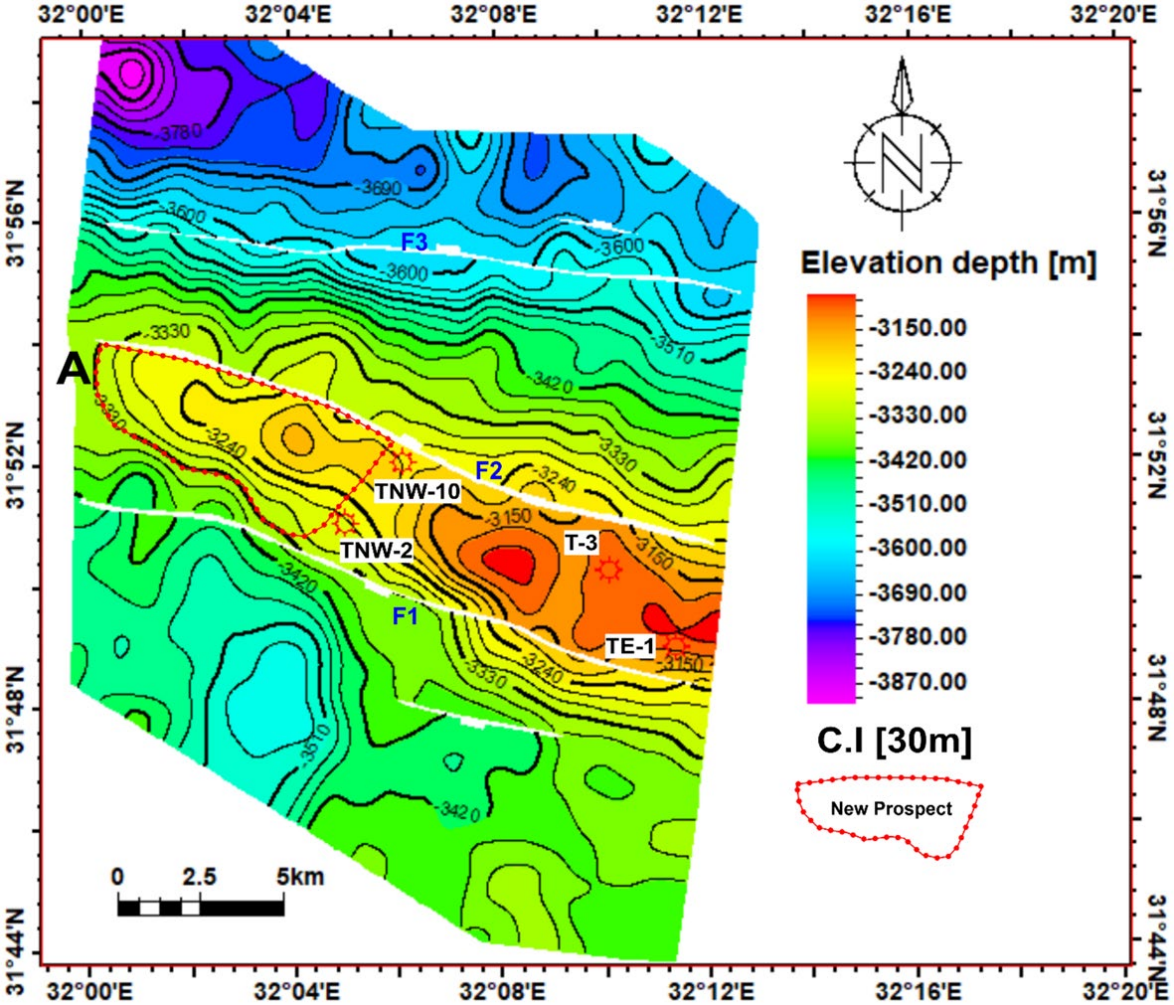
Depth structure contour map of the top Sidi Salem Formation, showing fault-bounded horst and step-fault closures. The red polygon delineates the newly identified gas prospect in the upthrown block, representing a three-way dip closure with strong trapping potential.

The Temsah Field primarily relies on the Sidi Salem Formation as its main reservoir, as indicated by the depth structure contour map depicting the top of this reservoir (Fig 9). The depth of the Sidi Salem Formation can be found at depths ranging from 3150 m to 3870 m. The orientation of all faults is in the northwest to southeast direction. These clusters of normal faults merge to form horst, and step fault blocks. All drilling operations were carried out on the top side of the interpreted faults that affect the Neogene-Quaternary reservoirs in the offshore Nile Delta Basin. These fault closures exhibit beneficial properties, including well-defined sealing regions, which generate excellent circumstances for the ac-cumulation of oil reserves.

The structural framework of the East Mediterranean region is controlled by the interaction of three main fault trends: the northwest-southeast Misfaq-Bardawil fault, the northeast-southwest Qattara-Eratosthenes fault, and the east-west faults delimiting the Messinian salt basin. These faults, likely reactivated ancient structures, produce characteristic horst, graben, and stair-step fault blocks that have persisted since the Miocene, influencing overlying sedimentary formations^45^.

The observed structural style—particularly the interplay of horst blocks and step faults—is consistent with fault systems described by^1,37,48^, who reported NE–SW and NW–SE trending faults as key trapping mechanisms in the Nile Delta Basin. However, our results refine the structural mapping by resolving the precise geometry of the horst block and associated closures using high-resolution seismic interpretation. The petrophysical parameters obtained in this study are within the range reported by^19–25^ for Neogene formations in the Nile Delta, but the clear spatial correlation between facies distribution and reservoir quality identified in our work adds a new dimension to reservoir characterization in this area. Moreover, the GIIP estimate for the identified prospect demonstrates a substantially higher potential than previously reported for comparable fault-bounded traps in the Temsah Concession.

### Three-dimensional structural model

**Fig. 10:**
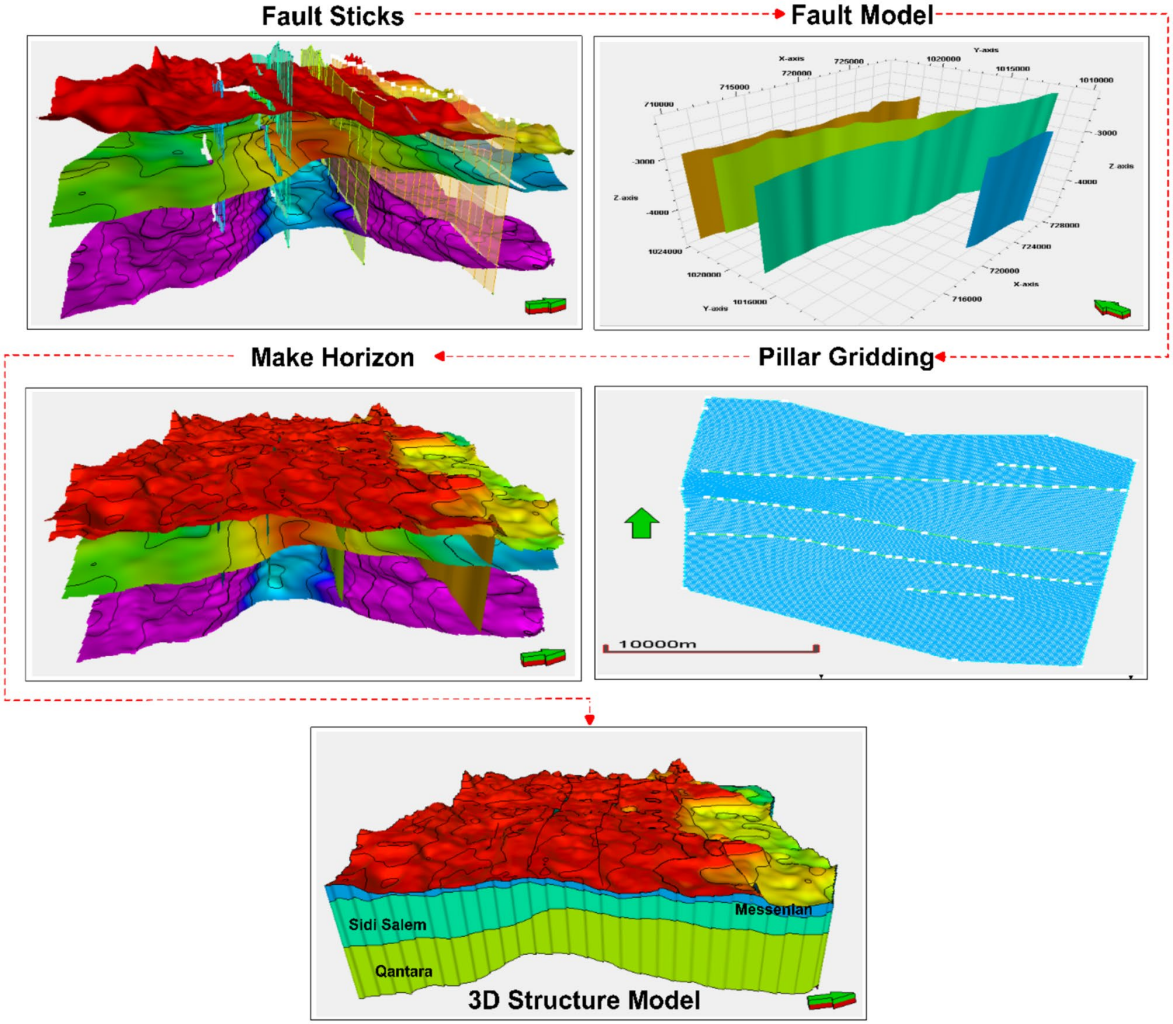
Main procedures of 3D structural model construction for the Temsah Field, including fault modeling, framework gridding, and horizon subdivision (Petrel, 2017). This framework served as the foundation for subsequent facies and property modeling.

After analyzing seismic data and creating structural maps, the next step was to develop a comprehensive structural model for the Temsah field. This model was created in three main stages using Petrel software Schlumberger 2017. Initially, the focus was on studying the fault system that affected the target formations being investigated. Then, a pillar gridding technique was used to establish a grid framework for the model. Finally, the horizons were identified and categorized, which enabled their division into stratigraphic horizons/units. This division helped in identifying structural impacts, variations in rock types, and changes in the petrophysical properties of the Sidi Salem reservoir (Fig 10). This approach provided a detailed understanding of the subsurface structure and offered valuable insights for reservoir characterization and analysis in the designated area.

**Fig. 11:**
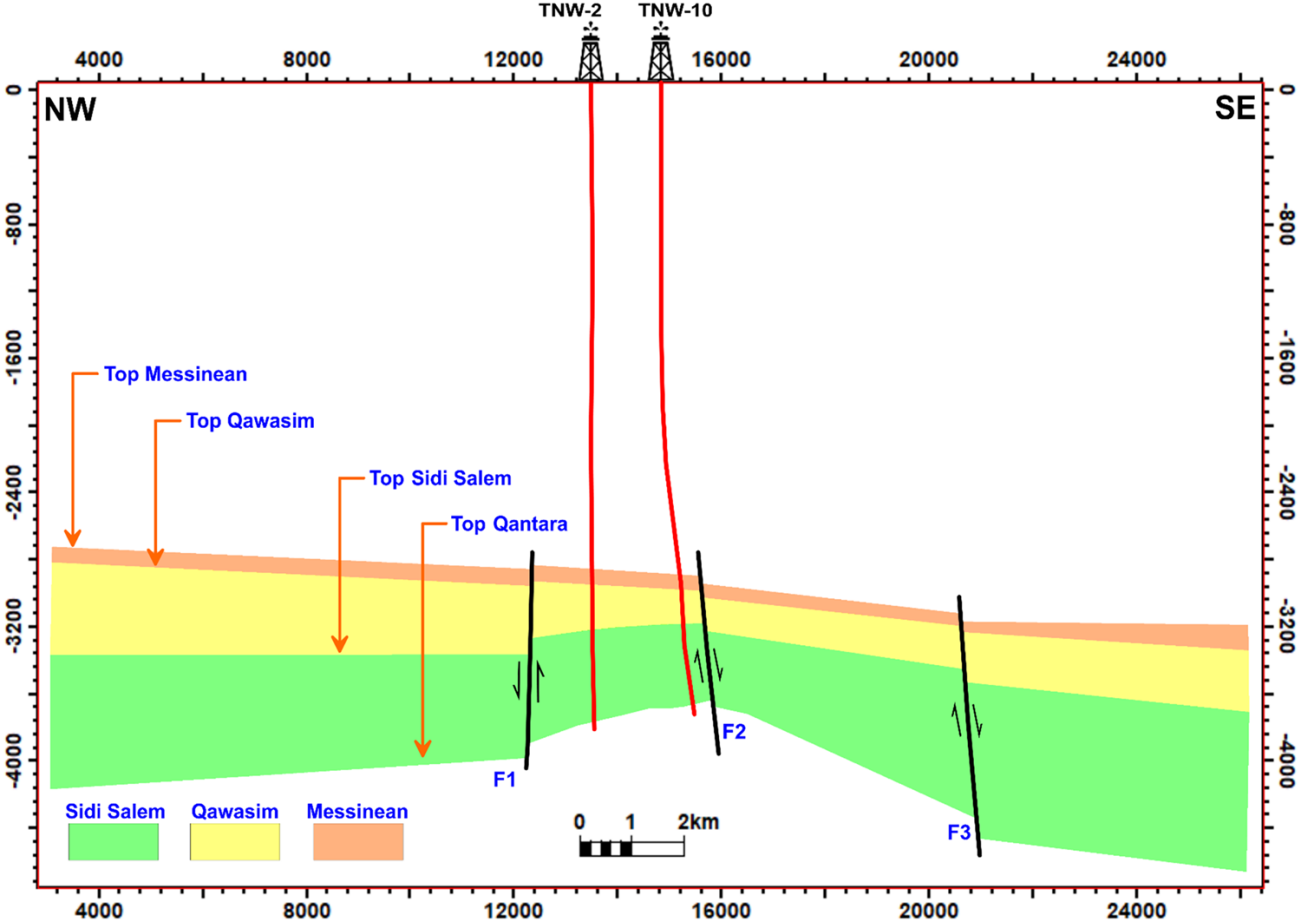
NW–SE structural cross-section through the Sidi Salem Formation, showing the influence of NE–SW and NW–SE trending faults. The section demonstrates how fault interactions create horst and step-fault blocks that act as primary hydrocarbon traps in the Temsah Field.

In Fig 11, a vertical structure cross-section is presented, illustrating how structures impact the Sidi Salem reservoir in the Temsah field located in the offshore Nile Delta Basin. The NE-SW structural cross-section reveals that the Sidi Salem Formation is affected by three normal faults. These faults exhibit orientations in the northwest-southeast and northeast-southwest directions. The interaction between these faults gives rise to geological features such as horst, and step fault blocks. It is worth noting that the study wells were strategically drilled near the upper displacement zones of these faults. These findings offer valuable insights into the structural behaviour and reservoir potential of the studied area, thereby assisting in the optimization of exploration and production strategies.

### Three-dimensional property model

The 3D static property model serves as a platform for integrating various types of data related to reservoirs, such as geological, petrophysical, and geophysical information. This model, as described by^78–83^ provides a detailed representation of the spatial distribution and variability of reservoir properties like porosity, permeability, and fluid saturation. By understanding the reservoir heterogeneity and identifying areas with different productivity levels, the model assists in making informed decisions regarding well placement and reservoir management strategies^84–87^. Upscaling the facies and petrophysical features of reservoir formations is crucial for improving computational efficiency, model representation, and reservoir management. When dealing with extensive reservoir simulations or field development plans, the complex calculations required for high-resolution facies and petrophysical data obtained from well logs can be computationally demanding. Therefore, upscaling allows for a more efficient portrayal of reservoir properties while retaining the essential features that affect fluid flow behaviour. The main petrophysical characteristics of the Sidi Salem Formation which is the main reservoir in the deep target are displayed in the Table 1.

**Table 1:** Petrophysical characteristics of the Sidi Salem reservoirs in Temsah field.

Well name	Reservoir	Net pay [m]	Effective porosity [%]	Shale content [%]	Water saturation [%]	Hydrocarbon saturation [%]
TNW-2	Sidi Salem	**62**	**21**	**25**	**25**	**75**
TNW-10		**120**	**24**	**19**	**23**	**77**
T-3		**68**	**34**	**8**	**21**	**79**
TE-1		**22**	**19**	**27**	**30**	**70**

m: meter, %: percent.

**Fig. 12:**
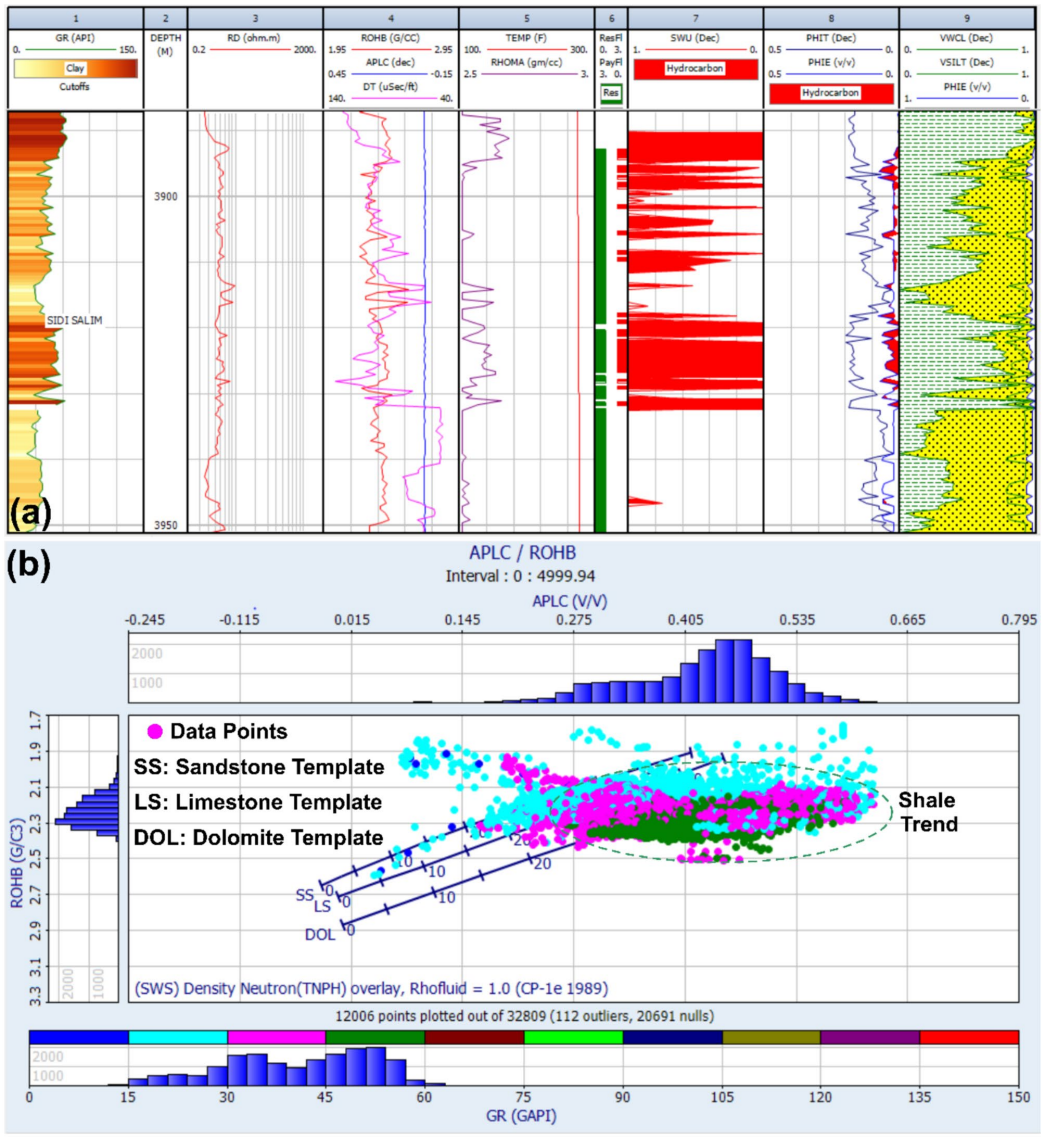
**a** Computer-processed interpretation of petrophysical parameters for the Sidi Salem Formation, including porosity, water saturation, and shale content. **b** Neutron–density cross-plot from the TNW-2 well with mineralogical template, illustrating lithological variations between sandstone and shale.

Fig 12 presents a vertical distribution of key petrophysical parameters within the Sidi Salem Formation, encapsulating vital insights across nine tracks essential for comprehensive reservoir evaluation. Beginning with the Gamma Ray log track, it delineates various rock types and shale content, crucial for identifying reservoir composition. Following is the depth track, ensuring precise location referencing for accurate analysis. The subsequent resistivity log track sheds light on formation electrical properties, aiding in pin-pointing potential hydrocarbon-rich zones. Meanwhile, the neutron/density log track provides insights into porosity and lithology variations within the reservoir, essential for understanding its structural integrity. Utilizing data from these tracks, the calculated temperature track assesses thermal conditions, influencing overall reservoir behaviour. The reservoir and pay flag track highlight zones with the highest hydrocarbon potential, pivotal for efficient reservoir management and optimization of production strategies. Simultaneously, the water saturation track estimates reservoir water content and its impact on hydrocarbon recovery, while the effective porosity log track offers insights into fluid flow dynamics, directly influencing reservoir productivity. Finally, the lithological identification track utilizes the neutron/density cross plot to discern major lithologies such as shale and sandstone, significantly impacting hydrocarbon storage capacity. In summary, Fig 12 provides a holistic overview of petrophysical parameters, lithological identification, and reservoir characteristics within the impactful Sidi Salem Formation, facilitating a nuanced understanding of its hydrocarbon potential crucial for informed decision-making in reservoir management and production optimization endeavours.

**Fig. 13:**
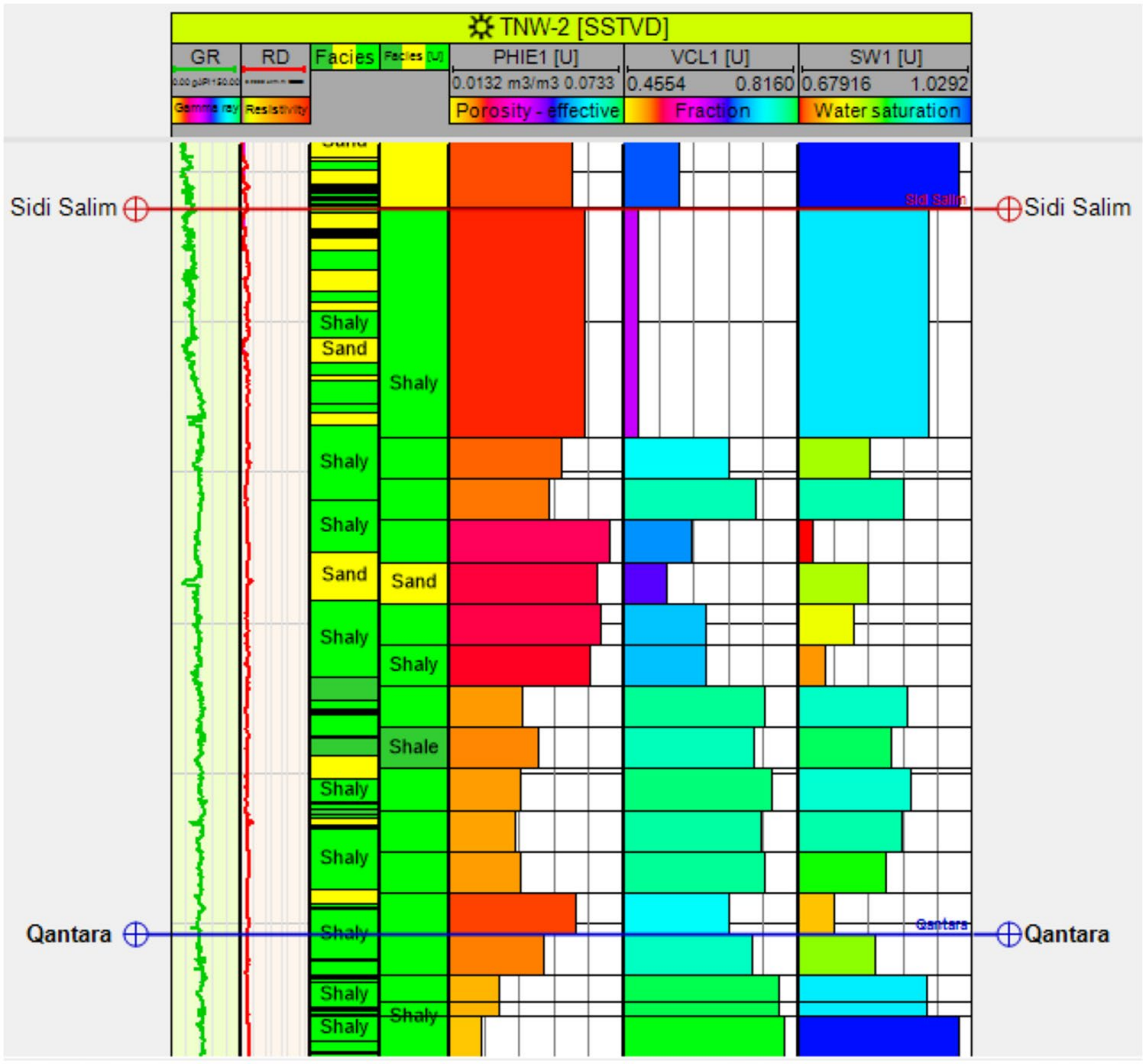
Vertical distribution and upscaling of facies and petrophysical properties within the Sidi Salem Formation (TNW-2 well). The figure highlights transitions between sandstone and shale facies, with corresponding changes in porosity, water saturation, and shale volume.

**Fig. 14:**
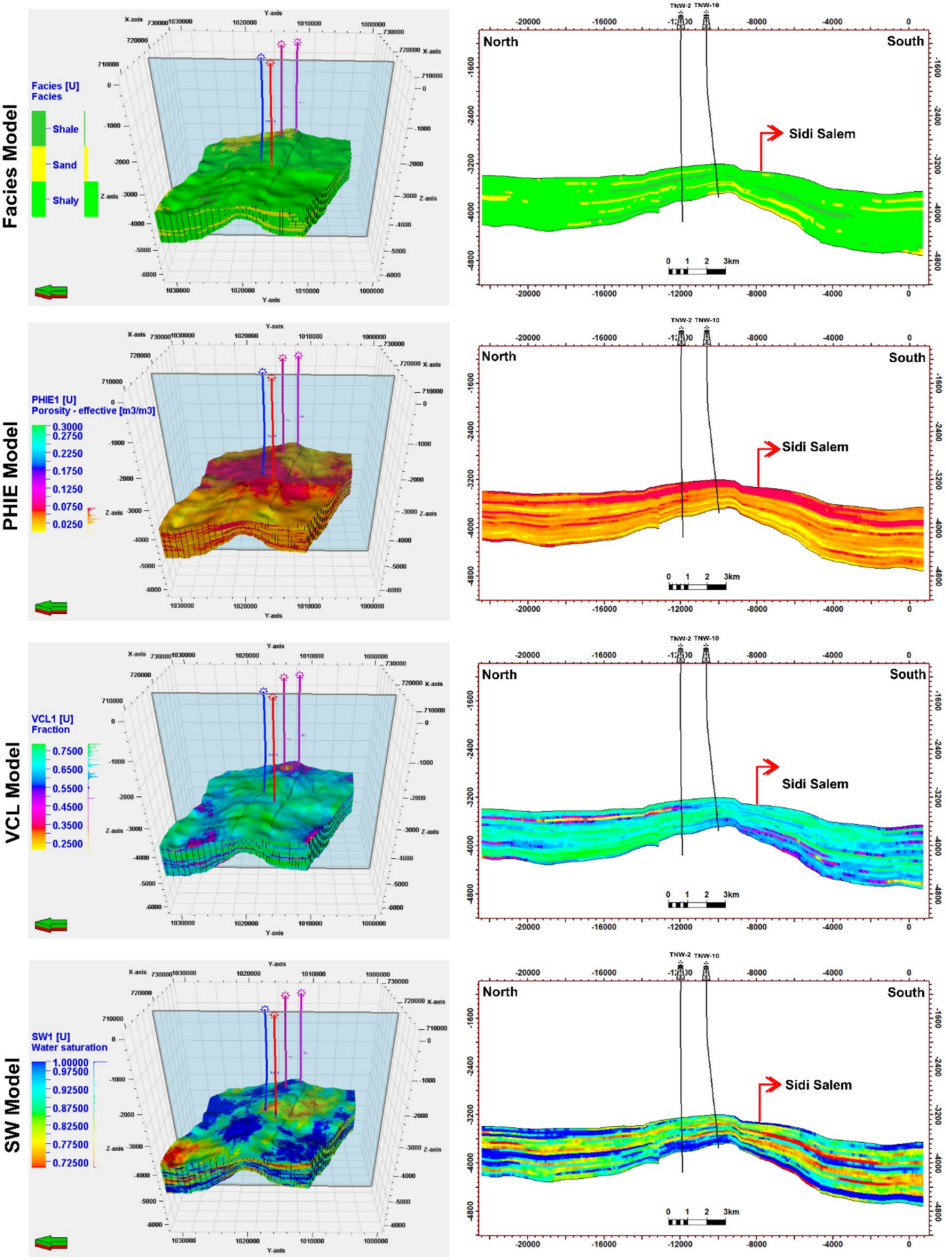
Three-dimensional facies and petrophysical models of the Temsah Gas Field (Petrel, 2017). The models display lateral and vertical variation in sandstone and shale facies, effective porosity, shale content, and water saturation, providing a comprehensive view of reservoir heterogeneity.

Fig 13 demonstrates the vertical variations in lithofacies types and petrophysical properties of the reservoir formations, as well as the efforts made to scale up these important elements. The illustration portrays distinct geological facies comprising sandstone and shale. The Sidi Salem Formation is characterized by the presence of both shale and sandstone facies. Furthermore, the figure vividly represents the vertical variability observed in upscaled petrophysical parameters such as effective porosity, clay content, and degree of water saturation. To assign lithofacies and rock property values to the cells intersected by drill holes, the subsurface structural and stratigraphic setting of the Sidi Salem Formation were considered in the property models. Well-log data was used for lithofacies interpretation, where the logarithm of each interpreted sediment surface was differentiated based on two major lithofacies: sandstone and shale. Three-dimensional static models for lithofacies were created using Petrel software Schlumberger 2017, as shown in Fig 14. To capture small-scale vertical variations in detail, the Upper sand and Sidi Salem reservoirs were divided into twenty stratigraphic horizons/units. The 3D property models for effective porosity, shale content, and water saturation were developed based on petrophysical features, as depicted in Fig 14. The facies model indicates that the Upper Sand primarily consists of sandstone facies with intercalated shale, while the Sidi Salem Formation is predominantly composed of shale with intercalated sandstone.

**Fig. 15:**
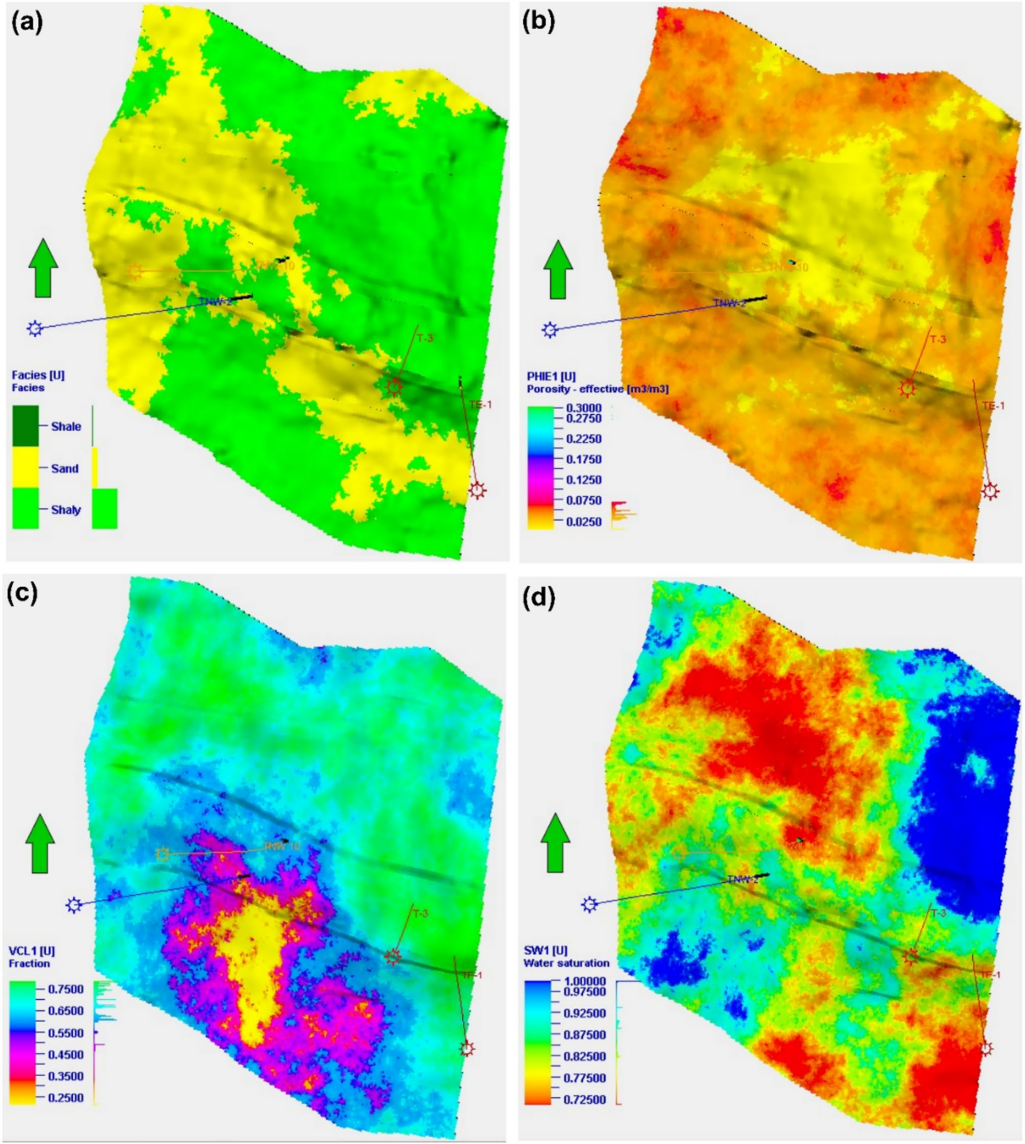
Selected productive layer of the Sidi Salem reservoir. (**a**) Facies distribution showing sandstone channels within shale-dominated intervals. (**b**) Effective porosity map identifying high-quality reservoir zones. (**c**) Shale content distribution highlighting heterogeneity. (**d**) Water saturation map indicating areas of enhanced hydrocarbon saturation (Petrel, 2017).

Fig 15 illustrates the spatial arrangement of rock facies within the Sidi Salem Formation, showcasing the presence of shale intercalated with sandstone facies. The figure also includes a distribution map of effective porosity, highlighting the reservoir’s shale composition and water saturation, these maps are created using Petrel software Schlumberger 2017. The Sidi Salem reservoir is predominantly characterized by the presence of shale facies, covering a significant portion of the region. However, the sandstone facies stand out with its distinct features, including a channel-like structure that extends from the southeastern to the northwestern side, as well as large sections of the western side. This unique appearance of the sandstone facies suggests its potential for storing hydrocarbons, indicating the possibility of significant oil and gas reserves in economically viable quantities.

The Sidi Salem reservoir stratigraphic horizons/unit has a consistent and significant rate of increase in most areas of the study region. However, it gradually diminishes towards the outskirts, particularly in the northern and eastern sections, while gradually intensifying in the central part of the study area. Conversely, the shale content in the study area exhibits a growth in the northern and eastern regions, while experiencing a large decline in the central, southern, and western parts of the Temsah area. As noted earlier, it is evident that the water content percentage of the Sidi Salem reservoir in the Temsah field experiences a substantial decline in the central area. This decrease persists in the northwestern and southeastern regions as well. Furthermore, it should be emphasized that this percentage exhibits a distinct rise in both the northeastern and southwestern regions. Acquiring this knowledge is crucial for precise analysis of reservoirs and improving tactics for finding hydrocarbon deposits. Ultimately, this will increase the success rates and economic feasibility of petroleum operations in the studied region. The variation in lithofacies and petrophysical parameters throughout the Nile Delta basin is primarily determined by the tectonic and structural characteristics of the area. The main geological formations that serve as barriers for hydrocarbon accumulation in this basin are directly linked to faulted blocks, which have a crucial influence on the distribution and concentration of hydrocarbons. Furthermore, the fault planes play a crucial role as significant pathways for the movement of hydrocarbons. In summary, the 3D static property model is crucial for assessing reservoirs as it provides a comprehensive and accurate representation of reservoir characteristics. It simplifies the process of comprehending and explaining reservoir characteristics, creating models and examining various scenarios, evaluating uncertainties, and improving methods to improve oil extraction. In the end, it facilitates the process of making informed judgments and implementing more effective reservoir management strategies.

### New prospect and reserve estimation

The Sidi Salem Formation exhibits substantial hydrocarbon saturation levels, reaching up to 79%, with effective porosity ranging from 19% to 34%. Shale content varies between 8% and 27%, while water saturation ranges from 21% to 30%, indicating high-quality sandstone reservoirs closely associated with source rocks and seals (Figs 9, 14-15)^71,84–87^.

Our integrated lithofacies and petrophysical analysis delineated sandstone and shale distribution, revealing high-quality reservoirs located within fault-bounded traps in the Temsah field. Depth contour maps of the Sidi Salem reservoir illustrate a three-way structural closure, indicating potential hydrocarbon accumulation (Fig. 9). This prospective zone, situated on the upthrown side of interpreted faults, is marked with a dashed red line in Fig 9 and represents a prime target for future exploration and development activities.

Based on the volume calculations, the estimated gas initially in place (GIIP) for this new prospect is approximately 5,332,660 BCF, alongside oil reserves of about 1,599,798 STB. These volumes demonstrate significant resource potential that can directly impact production strategies in the offshore Nile Delta’s Temsah gas field.

The results align with existing literature, highlighting the critical role of NE-SW and NW-SE trending normal faults in forming effective traps within the Nile Delta Basin. The interbedded sandstone and shale facies of the Sidi Salem Formation conform to deltaic depositional models, enhancing reservoir heterogeneity and potential^71^. The use of integrated seismic and well log data to construct a 3D static reservoir model follows best practices, confirming that detailed structural analysis and petrophysical characterization are vital for optimizing exploration and maximizing hydrocarbon recovery.

The outcomes of this study extend beyond the Temsah Field, offering practical insights applicable to other deltaic and structurally complex hydrocarbon provinces worldwide. The integrated approach combining high-resolution seismic interpretation, petrophysical evaluation, and 3D static reservoir modeling has proven effective in delineating fault-bounded traps and predicting reservoir heterogeneity in clastic successions. Similar geological complexities are observed in the Niger Delta Basin, one of the world’s most prolific hydrocarbon provinces, where syn-depositional growth faults, rollover anticlines, and shale diapiric play critical roles in hydrocarbon accumulation^85,86^. Studies in the Niger Delta^88^ have emphasized that understanding fault geometries and associated stratigraphic variations is essential for identifying prospective reservoirs and reducing exploration risks in such settings. The workflow and findings of this study can thus serve as a reference model for optimizing exploration strategies, especially in deltaic basins were complex fault systems and rapid facies changes control reservoir distribution and quality. This has significant implications for improving reservoir characterization and prospect evaluation in similar environments, supporting more efficient hydrocarbon development in regions like the Niger Delta, the Gulf of Mexico, and the Indus Basin.

## Discussion

This study demonstrates that an integrated workflow combining high-resolution seismic interpretation, detailed petrophysical evaluation, and advanced three-dimensional static modeling is essential for characterizing the structurally complex Temsah Gas Field. Each dataset provided distinct but complementary insights, and their integration enabled a more robust geological interpretation and hydrocarbon assessment.

High-resolution seismic data resolved the geometry of NE–SW and NW–SE trending normal faults, which form horst and step-fault blocks controlling reservoir compartmentalization. Fault dip values of 60°–80°, quantified from seismic dip measurements, directly influence trap dimensions and sealing capacity. The structural framework mapped in this study therefore provides a more accurate basis for hydrocarbon volume estimation than previous interpretations constrained by lower-resolution data^37,47^.

Petrophysical analysis of the four studied wells confirmed effective porosity values between 19% and 34%, shale content of 8–27%, and hydrocarbon saturation of 70–79%. These parameters, when spatially integrated with seismic interpretation, revealed that sandstone-rich intervals in the northwestern and southeastern sectors correspond to zones of enhanced reservoir quality. The consistency between depositional facies distribution and petrophysical properties underscores the role of both tectonic setting and sedimentary processes in reservoir heterogeneity^19,38–40,66^.

Three-dimensional structural and property modeling unified seismic and well-log interpretations into a coherent reservoir framework. Structural modeling using pillar gridding successfully reproduced the horst and step-fault architecture^64,65^, while property modeling distributed porosity, shale content, and water saturation across the grid^72,73,78,79^. This approach quantified reservoir heterogeneity, revealing channel-like sandstone facies aligned NW–SE, with porosity values commonly >30% and water saturation <25%. These intervals correspond to the most prospective hydrocarbon-bearing zones. The static model therefore provided a reliable platform for volumetric assessment and improved reservoir characterization.

The hydrocarbon volumetric analysis estimated a gas initially in place (GIIP) of approximately 5.33 TCF (5,332,660 BCF) and associated oil reserves of ~1.6 MMSTB for the newly identified fault-bounded closure. These volumes exceed previously reported estimates for comparable structures within the concession[13]and highlight the impact of integrating reprocessed seismic and quantitative petrophysical data into volumetric workflows. The volumetric calculation followed the industry-standard method[74]incorporating gross rock volume, porosity, net-to-gross ratio, water saturation, and formation volume factor, thereby ensuring reproducibility and minimizing uncertainty.

The results align with established tectono-stratigraphic models of the Nile Delta, where reactivated Miocene fault systems govern hydrocarbon entrapment^1,37,47^. However, the present study improves upon earlier work by incorporating dip values into structural interpretation, refining closure geometry, and explicitly linking reservoir quality to facies distribution. This level of resolution is critical for reducing exploration risk in fault-controlled reservoirs^19–21,66,68^.

Beyond the Temsah Field, the methodology has broader applicability to other deltaic and structurally complex basins such as the Niger Delta, Gulf of Mexico, and Indus Basin. In such provinces, growth faults, rollover anticlines, and shale diapirism similarly control reservoir architecture and fluid migration pathways^80–82,85,88^. Applying an integrated seismic–petrophysical–modeling workflow in these settings can improve prediction of reservoir heterogeneity, enhance volumetric accuracy, and optimize drilling strategies^71,84^.

## Conclusions

This study aimed to characterize the Neogene–Quaternary formations of the offshore Temsah Gas Field in the northeastern Nile Delta Basin, with a focus on the Sidi Salem Formation. The primary objectives were to integrate seismic and well-log data to delineate structural and stratigraphic features, evaluate the hydrocarbon potential, and construct a 3D static geological model to support exploration and development planning.

- The Temsah field exhibits a structurally complex setting dominated by NE–SW and NW–SE trending normal faults, which play a critical role in hydrocarbon entrapment.
- Depth mapping of the Sidi Salem Formation revealed a horst fault block with a three-way dip closure and an associated step normal fault forming the primary gas trap.
- Petrophysical analysis indicated effective reservoir thicknesses ranging from 22 to 120 m, porosity between 19% and 34%, and hydrocarbon saturation from 70% to 79%.
- Integration of data from four wells and twenty-nine 2D seismic lines enabled precise horizon and fault interpretation, reducing uncertainties in structural mapping.
- 3D facies and petrophysical models identified higher sandstone concentrations in the northwestern and southeastern sectors, correlating with favorable reservoir quality parameters.
- A new highly prospective drilling location was identified with an estimated GIIP of approximately 5,332,660 BCF, demonstrating substantial potential for enhancing gas production.

The integrated methodology applied in this study has proven effective in resolving structural complexity, improving reservoir characterization, and guiding exploration in fault-controlled deltaic reservoirs. These findings provide a robust basis for optimizing field development strategies in the Temsah Gas Field and offer a transferable workflow for similar structurally complex settings.

## Data Availability

The datasets generated during and/or analysed during the current study are available under request from the corresponding author [Mohamed Reda] through the contacted Email: mohamedreda.88@azhar.edu.eg.
